# Employers’ perception of the costs and the benefits of hiring individuals with autism spectrum disorder in open employment in Australia

**DOI:** 10.1371/journal.pone.0177607

**Published:** 2017-05-18

**Authors:** Melissa Scott, Andrew Jacob, Delia Hendrie, Richard Parsons, Sonya Girdler, Torbjörn Falkmer, Marita Falkmer

**Affiliations:** 1 School of Occupational Therapy and Social Work, Curtin University, Perth, Western Australia, Australia; 2 Cooperative Research Centre for Living with Autism (Autism CRC), Brisbane, Queensland, Australia; 3 School of Public Health, Curtin University, Perth, Western Australia, Australia; 4 School of Pharmacy, Curtin University, Perth, Australia; 5 Rehabilitation Medicine, Department of Medicine and Health Science (IMH), Linköping University, Linköping, Sweden; 6 School of Education and Communication, CHILD programme, Institute of Disability Research, Jönköping University, Jönköping, Sweden; King's College London, UNITED KINGDOM

## Abstract

Research has examined the benefits and costs of employing adults with autism spectrum disorder (ASD) from the perspective of the employee, taxpayer and society, but few studies have considered the employer perspective. This study examines the benefits and costs of employing adults with ASD, from the perspective of employers. Fifty-nine employers employing adults with ASD in open employment were asked to complete an online survey comparing employees *with* and *without* ASD on the basis of job similarity. The findings suggest that employing an adult with ASD provides benefits to employers and their organisations without incurring additional costs.

## Introduction

Although previously described in the Diagnostic and Statistical Manual of Mental Disorders, fourth edition (DSM-V) [1], the terms Asperger syndrome and pervasive developmental disorder not otherwise specified (PDD-NOS) are now considered under the broader diagnosis of Autism Spectrum Disorder (ASD) as outlined in the DSM-V [2]. ASD represents a distinct category of developmental disabilities, characterised by difficulties in social interaction and communication, and restricted or repetitive behaviours [2]. The term ASD is among one of the preferred terms by the autism community and professionals [3]. For the purpose of this article, the term ASD will be used throughout to represent individuals on the autism spectrum, who do not have an intellectual disability, working in open employment, and acknowledges that the participants in the current study did not represent the whole autism spectrum.

Work is a source of economic independence with many benefits beyond those of financial gain [4], offering a sense of accomplishment and competence, providing structure, opportunities for socialisation, facilitating contribution to society and less reliance on government funding [5–7]. For adults with ASD the motivation for engaging in employment is no different to those of the general working population. Every individual *with* and *without* a disability has the right to work, to freely choose their employment, to work in just and favourable conditions and to be protected against unemployment [8]. Australia has among the lowest rates of employment of individuals with disability in the Organisation for Economic Co-operation and Development (OECD), with adults with ASD underrepresented in employment even in comparison to other disability groups [6, 9]. In Australia, the labour force participation rate for adults with ASD is 42% in comparison to 53% of all individuals with disabilities, and 83% for individuals without disabilities [10, 11]. However, Australian labour force participation rates for adults with ASD are high compared to other countries including the United Kingdom with only 15% of adults of working age with ASD in full-time paid employment [12, 13] and similarly in the United States where only 11% of adults with ASD are reported to be competitively employed [14, 15].

The core characteristics associated with ASD often result in adults with ASD confronting difficulties finding and securing employment [16, 17]. While the specific difficulties experienced by adults with ASD in obtaining employment may vary [18], they commonly include: promoting themselves in an interview, difficulty adjusting to new work environments and routines (including sensory sensitivities in the workplace), remembering and following instructions, planning and multi-tasking, communicating effectively and socially interacting with co-workers [16, 18–20]. In contrast, adults with ASD may perform well in job tasks that require systematic information processing, a high degree of accuracy in visual perception, precise technical abilities, increased concentration for long periods of time and a high tolerance for repetitive tasks [18, 21, 22]. Employees with ASD may also possess other qualities attractive to employers, including trustworthiness, reliability, integrity, attention to detail and low absenteeism [19, 21, 23]. In an evaluation of 38 employers’ expectations of job performance, employers rated punctuality, willingness to work hard and attendance as the most important aspects of a job [24, 25]. Despite many adults with ASD demonstrating these particular strengths once employed, low employment rates suggest that other factors are influencing their employability [21].

A variety of external factors have been proposed as barriers to successful employment for adults with ASD, including a lack of specific vocational support services, traditional job application and interviewing processes, and limited workplace accommodations [26–29]. Another noted barrier to employment has been employer attitudes toward hiring and supporting individuals with disabilities [30–33]. While in general employers hold positive attitudes toward people with disabilities, when confronted with the process of hiring an individual with a disability many employers appear conflicted and reluctant to do so [32, 34], perceiving the costs as outweighing the benefits [35]. This belief may be underpinned by misconceptions or lack of knowledge regarding disability [36], such as the assumption that hiring individuals with a disability such as ASD, will incur higher costs due to poorer productivity [24]. Until this valid concern is addressed, it is likely that potential employers will continue to show reluctance in employing adults with ASD [37].

While the benefits and costs of both competitive and supported employment for adults with ASD has been examined from the perspective of the employee, taxpayer and society [13, 38–43], a paucity of literature attempts to describe this from the perspective of the employer [44]. In 2002, a cost-accounting methodology was developed by Cimera [45] identifying variables (e.g., supervision, employee turnover and worker’s compensation claims) that are likely to influence employers’ hiring decisions of both employees *with* and *without* disabilities. However, this methodology did not account for discrepancies in job position between employees [46]. To address this issue, Cimera [45, 46] proposed a “matched sample” framework whereby employees *with* and *without* disabilities are matched based on similar job titles, duties and responsibilities and then compared across work variables. For the purpose of this study the following work variables were of interest: workplace performance, supervision and training, and accommodations. Employer experiences of employing adults with ASD were also explored. Guided by Cimera’s framework, the primary aim of this study was to objectively examine the benefits and costs of employing adults with ASD from the perspective of the employer.

## Methods

### Participants

A survey was distributed nationally to approximately 250 Australian organisations with 59 employers of adults with ASD completing it. From the sample, approximately 19% were from micro organisations (<5 employees), with 23% from small organisations (5–19 employees), and 57% from medium (20–199 employees) to large sized organisations (200+ employees) (Table 1). Fifty-one percent of organisations recruited employees with ASD through disability employment service (DES) providers, while 49% of organisations recruited independently. The industry distribution was broad, with health care and social assistance (26.3%), retail trade (15.8%) and education and training (10.5%) being most prevalent, and representative of the size and industry type of Australia generally [47].

**Table 1:** Characteristics of employers and organisations.

	*n*	%
Industry^1^		
Health care and social assistance	15	26.3
Retail trade	9	15.8
Education and training	6	10.5
Professional, scientific and technical services	5	8.8
Accommodation and food services	4	7.0
Manufacturing	4	7.0
Other services	4	7.0
Information, media and telecommunications	2	3.5
Rental, hiring and real estate	2	3.5
Public administration and safety	2	3.5
Other	4	7.0
Main client base^1^		
Local community	24	41.4
State-wide	16	27.6
Nationwide	11	19.0
International	7	12.1
Number of full-time employees^1^		
<5	10	19.2
6–10	4	7.7
11–20	8	15.4
20–100	14	26.9
>100	16	30.8

^1^Excludes missing cases

### Procedure

Recruitment of participants occurred via two pathways; 1) with the assistance of autism and not-for-profit disability organisations and DES providers, and 2) cold calling multiple businesses and organisations Australia-wide. The initial recruitment process depended heavily on the assistance from autism organisations and DES providers contacting their employer database listed as employing individuals with ASD. Many DES providers were reluctant to share employer details due to the sensitive nature of their relationship and concern for overloading an already time-poor population, resulting in a poor response rate over a 3-month period (*n* = 12). To accommodate for this low response rate, two additional research assistants were hired to begin cold calling businesses and organisations, which were selected based on the following criteria: a) industry and; b) business size, particularly large organisations (including number of additional locations). All respondents were invited to participate if they were employing at least one adult with ASD, who met the DSM-IV criteria for Asperger Syndrome (AS)/High Functioning Autism (HFA) only. Employees were required to be over the age of 18 years and working in open employment for a period of at least 6 months, in full-time, part-time or casual positions.

Prior to completing the survey, respondents were required to match their employee *with* ASD (n = 59) to two employees *without* ASD (n = 96). According to the “matched sample” methodology proposed by Cimera [37, 46], employees were matched on the basis of job similarity within the workplace (perform the same job duties, require the same skills and work capacity) and compared across cost-accounting variables (wages, hours worked per week, supervision, accommodations, and wage subsidies). This matched-sample approach enabled comparisons between the two groups as the employers reported on employees that they perceived as comparable, and ensured that the difference between the two groups were likely attributable to disability status rather than job responsibilities and skills. The chosen methodology also necessitated a reliance on employees declaring to their employers that they had AS/HFA. When a match could not be made between employees *with* and *without* ASD the survey was only completed for the employee with ASD. A post hoc power calculation indicated that a sample of the obtained size was adequate to identify moderate difference in the main outcomes between employees *with* and *without* ASD (effect size = 0.4), with 80% power and α = 0.05.

### Survey development

The survey addressed the work variables of interest in the following four categories: 1) employer characteristics, 2) employer experiences of employing an adult with ASD, 3) work conditions and, 4) employment costs. Development of the survey was informed by current research literature and consultation with representatives from disability employment service providers and researchers with experience in ASD. Following feedback on the survey from a reference group comprised of industry experts, employers of adults with ASD, employment co-ordinators from disability employment service providers and researchers, a full edited version of the survey was piloted with two employers of adults with ASD and finalised (S1 Appendix). The survey was administered online using the Qualtrics platform [48], with a paper version available on request and taking approximately 45 minutes to complete.

### Data analysis

This study explored the perceived costs from the employers’ perspective. The cost values were based on respondents’ perceptions and estimates for the fiscal year 2015 in Australian dollars. Statistical analysis was conducted using the SAS version 9.2 software [49]. Descriptive statistics were used to summarise employer characteristics and experiences employing an adult with ASD. Using ordinal regression and Chi-square statistics, work performance was compared between groups for *‘above and below standard’* versus *‘meets standard performance’*. Regression models were used to compare the cost-related variables including hourly wage (full-time and part-time employees were grouped respectively and calculated separately), weekly supervision costs, total weekly costs and training costs between employees *with* and *without* ASD. Correlations between responses from the same employer were taken into account as a random effect in the models (the SAS Mixed procedure).

The weekly cost to the employer for each employee was estimated as follows:

Cost($AUS)=(hourlywage)x(hoursofwork)x(1-subsidyproportion)+(costofweeklysupervision)

Following convention, a p-value <0.05 was taken to indicate a statistically significant association in all tests.

### Ethical considerations

An information letter was sent to employers, briefly outlining the purpose of the study and inviting them to participate. Completed online or returned surveys were taken as consent to participate in the study. Ethical approval was obtained from the Curtin University Human Research Ethics Committee (HR37/2015) in Perth, Western Australia.

## Results

### Employer characteristics

#### Employment of adults with ASD

Forty-five percent of the organisations employed more than one employee with ASD, sixty percent had previously employed an adult with ASD, and more than three quarters had been employing adults with ASD for two or more years (Table 2). In approximately half of the organisations, the employee with ASD had been recruited through a DES provider.

**Table 2:** Current and previous employment of adults with ASD.

Factors	*n*	%
Number of employees with ASD in the organisation^1^		
1	29	54.7
2	6	11.3
3–5	11	20.8
≥6	7	13.2
Organisations previously employing adult with ASD		
Yes	27	60.0
No	18	40.0
Number of years employing adult with ASD^1^		
<1	7	13.2
1–3	17	32.0
4–8	16	30.2
≥9	13	24.5
Organisations recruiting employee with ASD through a disability employment service provider^1^		
Yes	26	51.0
No	25	49.0

^1^Excludes missing cases

### Employer experience employing an adult with ASD

Employer experiences of employing an adult with ASD was considered in relation to workplace impact and workplace performance of the employee with ASD.

#### Impact in the workplace

Reasons for employing an adult with ASD. Participants reported several reasons for employing an adult with ASD within their organisations, (Table 3). Contact by an agency (e.g., DES provider), and/or a policy of corporate social responsibility accounted for fifty percent of organisations’ responses, followed by the employee being the best candidate for the job at interview. Other reasons included the employee being previously known to the employer or the employee’s family approaching the employer directly.

**Table 3:** Reasons for employing adult with ASD in the organisation.

Reasons^a^	*n*	%
Employer contacted by an agency	19	32.2
Organisational policy of corporate social responsibility	12	20.3
Best candidate for the job at interview	9	15.3
Previously known to the employer	7	11.9
Employee with ASD approached the employer directly	7	11.9
Family inquiry made directly to employer	6	10.2
Other reasons	22	37.3

^a^Multiple responses allowed

Interactions in the workplace. Over fifty percent of employers reported friendly mixed exchanges between employees *with* and *without* ASD, during both work and out of work conversations. In contrast, around one fifth reported that employees with ASD struggled with interacting with co-workers. Across this spectrum of diverse interactions employers reported relatively limited interaction, with a fifth of employees with ASD reportedly only interacting with a few co-workers, with slightly more than ten percent of conversations being solely work-related and/or restricted to daily greetings between co-workers (Table 4).

**Table 4:** Interaction between the employee with ASD and co-workers.

Type of interaction^a^	*n*	%
Friendly mixed exchanges of both work and out of work conversations	33	55.9
Employees only interacts with a few of the other workers	12	20.3
Solely work-related conversations between workers	7	11.9
Restricted to greetings between workers	7	11.9
Employees struggles with interaction with other workers	11	18.6
Not applicable	3	5.1

^a^Multiple responses allowed

Impact of employee with ASD in the workplace. Overall, the impact of having an employee with ASD in the workplace was overwhelmingly positive (Table 5), particularly in regard to increasing awareness of ASD, and in promoting a culture of inclusion. Employees with ASD also contributed new creative and different skills to the work environment and positively impacted on workplace morale. Some of the less positive impacts of employees with ASD were the need for continuous supervision, instances of miscommunication with other employees and workplace conflict resulting from colleagues’ lack of ASD specific knowledge and staff training. Despite some of these less positive impacts, no employers indicated that employing an adult with ASD resulted in reduced productivity.

**Table 5:** Impact of having an employee with ASD in the workplace.

Impact^a^	*n*	%
Increased awareness regarding people with ASD in the workplace	35	59.3
Positive adaption in workplace culture to include and make the employee with autism feel part of the team	33	55.9
New creative and different skills have been brought to the workplace	19	32.2
Improvements in workplace morale	14	23.7
Lack of ASD-specific knowledge often leads to miscommunication between colleagues	7	11.9
Need for continuous workplace supervision of this employee has increased workload for other staff	10	16.9
Lack of ASD-specific staff training has resulted in an increase in workplace conflict between colleagues	5	8.5
Decreased productivity by the team	0	0.0
Other impacts	8	13.6
Not applicable	2	3.4

^a^Mulitple responses allowed

Employer recommendation. The majority of employers reported that they would recommend employing an adult with ASD to a business associate, with very few responding that they would not do so. In addition, more than fifty percent of employers indicated they would employ another adult with ASD if the current employee with ASD left the workplace (Table 6).

**Table 6:** Employer opinions on employing an adult with ASD.

Factors	*n*	%
Employers who would recommend employing an employee with ASD		
Yes	39	66.1
No	2	3.4
Possibly	18	30.5
Replacement of employee with ASD if this person left the workplace		
Similar worker with ASD	31	52.5
Worker without ASD	5	8.5
Would not be replaced	4	6.8
Not sure	19	32.2

#### Workplace performance

Employee requirements for workplace performance. Employees *with* and *without* ASD were compared on the extent to which they met standard requirements for good workplace performance. The responses indicated employees with ASD performed at an above standard level in regard to attention to detail, work ethic and quality of work (Table 7). However, employees with ASD performed at a below standard level in regard to flexibility and following instructions. Responses for completion of work tasks on time revealed an interesting pattern with employees with ASD more likely to perform both at above and below standard levels.

**Table 7:** Extent to which employees met requirements for good workplace performance.

Characteristics	Standard of work^1^		
	Above n(%)^2^	Meets n(%)^2^	Below n(%)^2^
Flexibility			
No ASD	29 (30.2)	59 (61.5)	8 (8.3)
ASD	10 (19.6)	27 (52.9)	14 (27.5)
Attends to detail			
No ASD	18 (19.0)	67 (70.5)	10 (10.5)
ASD	28 (54.9)	19 (37.3)	4 (7.8)
Completes work on time			
No ASD	20 (21.30	67 (71.3)	7 (7.5)
ASD	19 (37.3)	24 (47.1)	8 (15.7)
Follows instructions			
No ASD	28 (29.8)	62 (66.0)	4 (4.3)
ASD	14 (27.5)	30 (58.8)	7 (13.7)
Work ethic			
No ASD	28 (30.1)	58 (62.4)	7 (7.5)
ASD	36 (70.6)	12 (23.5)	3 (5.9)
Productivity			
No ASD	23 (24.5)	63 (67.0)	8 (8.5)
ASD	17 (34.0)	26.(52.0)	7 (14.0)
Quality of work			
No ASD	24 (25.9)	64 (68.8)	5 (5.4)
ASD	21 (41.2)	27 (52.9)	3 (5.9)

^1^Excludes missing cases

^2^ Percentages of responses within employee type.

Standards of workplace performance. Employees *with* ASD had significantly better attention to detail in work tasks and in their work ethic compared to employees *without* ASD, however they were also significantly less flexible when completing work tasks. There were no significant differences between employees *with* and *without* ASD in their ability to follow instructions, their productivity and quality of work. Completing work on time yielded mixed results. While the majority of employees *without* ASD met the standard for completing work on time, there was a greater proportion of employees *with* ASD both above (p<0.0145) and below standard (p<0.0417) in their workplace, resulting in significant differences in both directions (Table 8).

**Table 8:** Multinomial regression analysis of employees meeting requirements for good workplace performance^a^.

Outcome	Employee	Odds ratio	95% confidence interval	p-value
Flexibility				
Below standard	No ASD	1 (reference)		
	ASD	3.82	1.43–10.20	0.0074
Above standard	No ASD	1 (reference)		
	ASD	0.75	0.32–1.77	0.5145
Attends to detail				
Below standard	No ASD	1 (reference)		
	ASD	1.41	0.40–5.01	0.5945
Above standard	No ASD	1 (reference)		
	ASD	5.49	2.51–11.98	<0.0001
Completes work on time				
Below standard	No ASD	1 (reference)		
	ASD	3.19	1.05–9.74	0.0417
Above standard	No ASD	1 (reference)		
	ASD	2.65	1.21–5.80	0.0145
Follows instructions				
Below standard	No ASD	1 (reference)		
	ASD	3.62	0.98–13.32	0.0532
Above standard	No ASD	1 (reference)		
	ASD	1.03	0.48–2.24	0.9340
Work ethic				
Below standard	No ASD	1 (reference)		
	ASD	2.07	0.47–9.18	0.3376
Above standard	No ASD	1 (reference)		
	ASD	6.21	2.81–13.75	<0.0001
Productivity				
Below standard	No ASD	1 (reference)		
	ASD	2.12	0.70–6.45	0.1853
Above standard	No ASD	1 (reference)		
	ASD	1.79	0.82–3.89	0.1409
Quality of work				
Below standard	No ASD	1 (reference)		
	ASD	1.42	0.32–6.38	0.6452
Above standard	No ASD	1 (reference)		
	ASD	2.07	0.99–4.34	0.0528

^a^Proportional odds not assumed

### Work conditions

Employees with and without ASD were grouped respectively into full-time or part-time employment and each group was analysed separately account for work basis differences. Comparison of work profiles and conditions for employees indicate that employees *with* ASD were more likely to be employed on a part-time basis than employees *without* ASD (p<0.0414) (Table 9). No statistically significant differences between groups were found for level of supervision, modifications to the work environment or workplace training.

**Table 9:** Variables used to calculate weekly costs.

Variable	No ASD	ASD	Total	Tests of association
Work basis				p = 0.0414^1^
Full-time (FT)	42 (47.2)	14 (28.0)	56	
Part-time (PT)	21 (23.6)	22 (44.0)	43	
Casual	24 (27.0)	12 (24.0)	36	
Contract	2 (2.3)	2 (4.0)	4	
Missing	7 (7.3)	1 (2.0)	8	
Supervision required				*x*^2^_(1)_ = 3.3; p = 0.0680
Yes	47 (49.0)	33 (64.7)	80	
No	49 (51.0)	18 (35.3)	67	
Modifications required				p = 0.3745^1^
Yes	7 (7.3)	6 (11.8)	13	
No	89 (92.7)	45 (88.2)	134	
Training required				*x*^2^_(1)_ = 1.2; p = 0.2659
Yes	53 (55.2)	33 (64.7)	86	
No	43 (44.8)	18 (35.30	61	

^1^p-value calculated using Fisher’s Exact test

### Employment costs

Calculations of hourly wage for employees *with* and *without* ASD were based on the 112 employees with available data (Table 10). Hourly wages for employees *with* ASD was only marginally lower than those *without* ASD (difference of $1.65). No significant differences between employees *with* and *without ASD* were evident in the weekly supervision cost, weekly cost to the employers (both full-time and part-time) and costs related to workplace training.

**Table 10:** Comparison of employment costs for employees with and without ASD obtained from a random effects regression model.

Variable	Mean	95% confidence interval	p-value
Hourly wage			0.0248
No ASD	23.49	20.35–26.63	
ASD	21.84	18.61–25.07	
Weekly supervision cost			0.3373
No ASD	231.23	174.87–287.59	
ASD	255.76	198.26–313.26	
Weekly cost (full-time)			0.8916
No ASD	1033.10	836.41–1229.79	
ASD	1023.36	798.02–1248.70	
Weekly cost (part-time)			0.4436
No ASD	774.04	624.89–923.19	
ASD	734.06	593.45–874.68	
Cost of training			0.6362
No ASD	175.75	109.214.92	
ASD	184.21	116.85–251.56	

## Discussion

Understanding the impact of external factors influencing the employment of adults with ASD is imperative for closing the unemployment gap. One of the main external factors influencing employability is employer attitudes toward hiring people with a disability. The present study attempts to answer the fundamental question of whether hiring adults with ASD is a good business decision from the perspective of the employer by comparing the costs and benefits of employees *with* and *without* ASD.

Findings indicated that employees *with* ASD received a marginally lower hourly rate than their colleagues *without* ASD (difference $1.65). This is likely attributable to the underemployment of adults with ASD, who often work restricted weekly hours (<8 hours per week) or are in part-time roles, earning lower wages than employees *without* ASD [7, 16, 50]. In Australia, subsidies are widely available for employers employing individuals with a disability, including ASD, for financial assistance for payment of pro-rata wages, workplace modifications and services and as a financial incentive to ongoing employment [51]. It is likely that this lower hourly rate for employees with ASD is at least in part influenced by these subsidies. Although findings from this study have highlighted a gap in remuneration for employees with ASD they also indicated that while they may require some workplace modifications, supervision and training, there is no significant difference between them and their colleagues in regard to weekly employment, supervision and training costs. Previous research has suggested employer concerns related to hiring people with a disability are associated with increased costs for ongoing supervision, training and accommodation [35, 37, 40]. Although this data needs to be interpreted with caution due to its sample size, these findings suggest that employers do not incur additional costs when employing an adult with ASD over and above that associated with any new employee.

Another employer concern is that of productivity and workplace performance of employees with disabilities. In this study, employees with ASD demonstrated above standard workplace performance when compared to their counterparts with regard to increased attention to detail, work ethic and quality of work. These outcomes point to qualities which are attractive to employers and common among people with ASD, such as reliability, integrity and consistent accuracy in performance [16, 22]. Findings from this study revealed that the employees *with* ASD were at least as productive as employees *without* ASD, challenging the assumption that hiring an individual with ASD, results in an employee with lack of work skills and reduced productivity [52, 53]. While recognising there are challenges associated with employing adults with ASD, such as following instructions, and flexibility and perseverating on work tasks [54], if not appropriately managed can potentially impact on productivity. Many of these challenges could be ameliorated by structuring and adapting work tasks, direct communication, and understanding individual support needs [19, 21, 55]. Should such strategies be implemented via approaches such as supervision, training and accommodations, our findings suggest that employers will incur no additional costs than any other employee, potentially reducing employer concerns of additional costs [56].

Favourable employer attitudes toward hiring individuals with disabilities is associated with larger (100+ employees) organisations and previous experience [36, 57]. Nearly a third of respondents in the current study were associated with large organisations suggesting that they were more likely to hire adults with ASD compared to medium or small organisations. This may be the result of large organisations having increased resources, less concern with the perceived “additional costs” for supervision, training and accommodations and an increased awareness and compliance with social corporate responsibility [36, 58]. Previous experience working with individuals with disabilities also positively influences future employment decisions [36], a finding supported in this study with 60% of respondents previously employing adults with ASD. Lastly, another factor contributing to favourable employer hiring decisions may be external support from a DES provider [36]. DES providers assist with recruitment, job placement, accommodations and ongoing support. Collaboration between employers and DES providers has been identified as a key component promoting positive employment outcomes for employees with a disability [33, 53, 59–61]. Fifty percent of respondents in this study were associated with and had recruited employees with ASD through a DES provider. These factors are likely to play an important role in successful employment of adults with ASD by reducing employers’ potential prejudices [36].

Lastly, findings from this study point to some additional widespread organisational benefits of employing an adult *with* ASD, which are difficult to quantify. Employers indicated the positive impact employing an adult with ASD had on the workplace culture, particularly the addition of new and creative skills, the increase in ASD awareness and a conscious positive shift in workplace inclusion. A diverse and inclusive workplace where employees feel valued, plays a critical role in work performance, productivity and job success of employees with disabilities [62]. Diversity and inclusion is also beneficial to organisational success [30], offering a competitive edge in creativity, enhancing relationships with the community and improving job retention.

### Limitations

There are several limitations to this study. Firstly, the relatively small sample size may not be completely representative of the broader population of employers of adults with ASD. Therefore, the cost component estimates may not reflect the general broader Australian context. However, the main comparisons in this study was between employees *with* and *without* ASD under each employer, and while the absolute costs and experiences may differ between this sample and the broader population, it is likely that the relative differences between employees *with* and *without* ASD would be in the directions shown in this study. Secondly, given the complex nature of this research, recruitment of employers was particularly difficult. Of the 250 employers approached to participate in this study, only a quarter responded and results should be interpreted with caution. Respondents could well have been those who had the most positive experiences, thereby being more likely to participate in this study. It is possible that the results would have differed if non-responding employers have chosen to complete the survey. Another explanation may be that 51% of respondents are supported by DES providers and due to the nature of their supportive relationship and the financial assistance provided, may have felt pressured to respond positively. Thirdly, despite persistent follow-up calls and emails, many respondents failed to complete the survey. The 60% attrition rate observed may be explained by the survey length and time required to complete the survey for the targeted employers, a group of participants who are well-known for being time-poor. Attrition may also have been the result of respondents being supervisors or managers of employees with ASD without direct access to employment cost information. In many organisations, it is the responsibility of the Human Resources department to manage confidential employee information. Lastly, this study relied heavily on employees declaring to their employer that they had ASD (AS/HFA), with no direct means of verifying the accuracy of these self-reports. While demographic information for employees with ASD (age, gender, presence of intellectual disability, severity and educational level) may have strengthened the methodological framework, it was not collected as this study focused on employer perspectives of the skills, abilities and benefits that adults with ASD as employees bring to the workplace and not on the characteristics of the condition, and it is likely that the addition of further questions would have further impacted on the response rate.

## Conclusions

Overall, this study found that employers do not incur additional costs when employing an adult with ASD over and above that associated with any new employee. Consequently, at the organisational level these results challenge employer attitudes that hiring adults with ASD may result in a loss of productivity and increased costs associated with workplace modifications and additional training and supervision. This study also identified the benefits of employing an adult with ASD such as significantly better attention to detail in work tasks and in their work ethic compared to employees *without* ASD. The addition of such strengths diversifies the workplace, potentially offering organisations a competitive edge [63]. Although this study may invite more questions, it is important that we continue to objectively address employer attitudes and concerns toward hiring and supporting employees with ASD, in order to improve their employment opportunities and strengthen and diversify the Australian workforce.

## Supporting information

S1 Appendix The benefits and costs to employers of employing an adult with High functioning autism survey. (DOCX)
