# Supplementary material for: Employers’ perception of the costs and the benefits of hiring individuals with autism spectrum disorder in open employment in Australia
Source: PLoS One. 2017 May 18;12(5):e0177607. doi: 10.1371/journal.pone.0177607 (PMC5436808; doi:10.1371/journal.pone.0177607)
Supplement: S1 Appendix — (DOCX) [file pone.0177607.s001.docx]

|  |
| --- |
| **The Benefits and Costs to Employers of Employing an Adult with High Functioning Autism Survey** |
|  |

|  |
| --- |

Dear Employer,

We need your help to understand the benefits and costs of employing an adult with autism. This survey will make an important contribution to assist current and future employment of individuals with autism in the workplace, as well as providing constructive information for prospective employers.

If you have any questions or would like more information, please contact one of the following researchers:

- Andrew Jacob- Email: andrew.t.jacob@student.curtin.edu.au or Mobile: 0439 855 904
- Melissa Scott- Email: melissa.scott@curtin.edu.au or Work Phone: 08 9266 5164
- Torbjorn Falkmer- Email: T.Falkmer@curtin.edu.au or Work Phone: 08 9266 9051

Thank you for your time and consideration.

**Consent Form**

See information sheet attached for more information.

**Consent to Participate:**

- I agree to participate in the study outlined to me
- I have been informed of and understand the purpose of the study
- I have had the opportunity to ask questions and they have been answered
- I understand that there are no known risks involved in the study
- I understand that participation is voluntary and that I can withdraw at any time without reason or consequence
- I have been informed that all personal information will be kept confidential and any identifiable information will not be used in published material
- I agree that the information I provide can be used in other studies and/or publications

Do you consent to participate in this study?

Yes

No, thank you

Signature:

________________________________________________________________

Name:

________________________________________________________________

Date:______________________________

**Instructions**

The questions in this survey relate to your employee(s) with autism spectrum disorders including high functioning autism, Asperger’s syndrome, any individual self-identifying as having autism. The information you provide will help improve employment practices and policies in regard to the employment of adults with autism.

The questions ask you to give answers on a specific employee with autism in your organisation (Employee A), as well as on two specific employees without autism who are matched on the basis of similar jobs (Employee B, Employee C).

If this matching process is not possible, due to jobs not being similar, matching on a basis of similar responsibilities, role or qualifications as close as possible is acceptable. If that is not possible, please fill in as much as you can about the employee with autism (Employee A).

Should you wish to inform employees that you are participating in this survey, you are encouraged to do so.

**Section 1: Experience Employing an Adult with Autism**

The purpose of this section is to collect information about your experience of being an employer of a specific adult with autism in your workplace (Employee A).

- 1. How many employees with autism does your organisation currently employ?

1

2

3

4

5

6+

I am not sure

- 1. The employee with autism was employed because: (Multiple selections allowed)

The individual was previously known to the employer

The employer was contacted by an agency

The employee with autism approached the employer directly for a job

This employee with autism was considered to be the best candidate in a job interview

The organisation’s policy of corporate social responsibility

A family inquiry was made directly to the employer

Other reasons (Please specify)

I am not sure

- 1. How would you describe the interactions between the employee with autism and fellow employees?

Friendly mixed exchanges of both work and out of work conversations

Solely worked related conversations between workers

Restricted to greetings between workers

The employee struggles with interaction with other workers

The employee only interacts with a few of the other workers

Not Applicable

- 1. How has having this employee with autism in your employment impacted your workplace? (Multiple options allowed)

Improvement of workplace morale

Increased awareness regarding people with autism in the workplace

Positive adaption in workplace culture to include and make the employee with autism feel

part of the team

New creative and different skills have been brought to the workplace

The lack of autism specific knowledge often leads to miscommunication between colleagues

Need for continuous workplace supervision of this employee has increased workload for

other staff

Lack of autism specific staff training has resulted in an increase in workplace conflict between

Colleagues

Decreased productivity by team

Other (Please specify)

Not Applicable

- 1. Would you recommend employing an employee with autism to a business associate?

Yes (Go to question 1.6.)

No

Possibly

If “possibly”, what other considerations does your decision depend on?

If “no”, what considerations influenced this decision?

- 1. Have other employees with autism been previously employed here?

Yes

No

I am not sure

- 1. If the employee with autism (Employee A) left the workplace, whom would you consider hiring?

Similar worker with autism

Worker without autism

Would not be replace

I am not sure

**These questions ask you to give answers on both an employee with autism in your organisation (Employee A), as well as two matching employees without autism who have similar jobs (Employee B, Employee C). If this is not possible, due to jobs not being similar, matching on a basis of similar responsibilities, role or qualifications as close as possible is acceptable. If that is not possible, please fill in as much as you can about Employee A.**

- 1. How would you describe this employee’s flexibility in the workplace on job tasks?

|  | Above Standard | Meets Standard | Below Standard |
| --- | --- | --- | --- |
| Employee A (autism) |  |  |  |
| Employee B |  |  |  |
| Employee C |  |  |  |

- 1. How would you describe the employee’s attention to detail?

|  | Above Standard | Meets Standard | Below Standard |
| --- | --- | --- | --- |
| Employee A (autism) |  |  |  |
| Employee B |  |  |  |
| Employee C |  |  |  |

- 1. How often does this employee complete their allocated amount of work on time?

|  | Above Standard | Meets Standard | Below Standard |
| --- | --- | --- | --- |
| Employee A (autism) |  |  |  |
| Employee B |  |  |  |
| Employee C |  |  |  |

- 1. How would you best describe this employee’s ability to follow instructions?

|  | Above Standard | Meets Standard | Below Standard |
| --- | --- | --- | --- |
| Employee A (autism) |  |  |  |
| Employee B |  |  |  |
| Employee C |  |  |  |

- 1. How do you feel about leaving this employee to interact with customers?

|  | Not Applicable | Completely independent | Interacts with customers, but supervisors are present | Requires some active supervision when dealing with customers | Not confident leaving them alone with customers |
| --- | --- | --- | --- | --- | --- |
| Employee A (autism) |  |  |  |  |  |
| Employee B |  |  |  |  |  |
| Employee C |  |  |  |  |  |

- 1. This employee’s work ethic is best described as:

|  | Above Standard | Meets Standard | Below Standard |
| --- | --- | --- | --- |
| Employee A (autism) |  |  |  |
| Employee B |  |  |  |
| Employee C |  |  |  |

- 1. Select the description that best describes the productivity of this employee:

|  | Above Standard | Meets Standard | Below Standard |
| --- | --- | --- | --- |
| Employee A (autism) |  |  |  |
| Employee B |  |  |  |
| Employee C |  |  |  |

- 1. Select the description that best describes the typical quality of work done by this employee:

|  | Above Standard | Meets Standard | Below Standard |
| --- | --- | --- | --- |
| Employee A (autism) |  |  |  |
| Employee B |  |  |  |
| Employee C |  |  |  |

Thank you, we really appreciate your insight.

**Section 2: Employer Information**

The purpose of this section is to collect background information about the workplace.

2.1. What is your role in the workplace?

Business owner

Manager

Supervisor

Other (Please specify)

2.2. Which industry best describes the main function of the organisation?

Accommodation and Food Services

Agriculture, Forestry and Fishing

Arts and Recreation Services

Construction

Education and Training

Electricity, Gas, Water and Waste Services

Financial and Insurance Services

Information Media and Telecommunications

Health Care and Social Assistance

Mining

Professional, Scientific and Technical Services

Public Administration and Safety

Rental, Hiring and Real Estate Services

Retail Trade

Transport, Postal and Warehousing

Wholesale Trade

Other (Please specify)

I am not sure

2.3. Which of the following best describes the main client base of the organisation?

Local Community

Statewide

Nationwide

International

Other (Please specify)

2.4. What is the approximate total number of employees in the organisation?

|  | 0-5 | 6-10 | 11-20 | 20-100 | 100+ |
| --- | --- | --- | --- | --- | --- |
| Full-time |  |  |  |  |  |
| Part-time |  |  |  |  |  |
| Casual |  |  |  |  |  |

2.5. Which of the following describe the approximate annual revenue of the business?

Under $100,000

$100,001-$500,000

$500,001-$1 million

$100,001 million-$500 million

$500,001 million-$1 billion

More than $1 billion

I do not know

2.6. For how many years have you been employing employees with autism?

Less than 1 year

1-2 years

2-3 years

4-8 years

9-12 years

13-16 years

17-20 years

More than 20 years

I do not know

2.7. Was the employee with autism recruited through a disability employment services provider?

Yes

No

I do not know

Thank you, we really appreciate your insight.

**Section 3: Work Conditions**

The purpose of this section is to collect information about the employment conditions for the three employees.

3.1. What is this employee's job description/title and key tasks (please list 3-5 key tasks)?

| **Job Title** | **Key tasks** |
| --- | --- |
| Employee A (autism): | 1.  2.  3.  4.  5. |
| Employee B: | 1.  2.  3.  4.  5. |
| Employee C: | 1.  2.  3.  4.  5. |

3.2. Approximately how long has this employee been employed at your organisation?

|  | < 6 months | 6-12 months | 13-18 months | 19-24 months | 25-30 months | 31-36 months | > 36 months |
| --- | --- | --- | --- | --- | --- | --- | --- |
| Employee A (autism) |  |  |  |  |  |  |  |
| Employee B |  |  |  |  |  |  |  |
| Employee C |  |  |  |  |  |  |  |

3.3. On what basis is this employee employed?

|  | Full-time | Part-time | Casual | Contract | Trial/  Probation |
| --- | --- | --- | --- | --- | --- |
| Employee A (autism) |  |  |  |  |  |
| Employee B |  |  |  |  |  |
| Employee C |  |  |  |  |  |

3.4. Do you use financial assistance (government assistance/funded programs) when paying wages

for this employee with autism?

Yes

No (Go to question 3.5.)

If “yes”, which financial assistance scheme do you use?

Supported Wage System

Wage Subsidy Scheme

Other (Please specify)

3.5. What percentage of the employee with autism wage is paid through the wage subsidy scheme?

|  | 1-5% | 6-10% | 11-20% | 21-40% | 41-60% | 61-70% | 71-90% | I do not know |
| --- | --- | --- | --- | --- | --- | --- | --- | --- |
| Employee A (autism) |  |  |  |  |  |  |  |  |

3.6. Please specify to whom the chosen three employees report:

|  | Supervisor | Manager | Peer | Business owner | Other |
| --- | --- | --- | --- | --- | --- |
| Employee A (autism) |  |  |  |  |  |
| Employee B |  |  |  |  |  |
| Employee C |  |  |  |  |  |

3.7 What is the average hours worked by the employee per week (excluding overtime)?

|  | <10 | 11-15 | 16-20 | 21-25 | 26-30 | 31-35 | >35 | I do not know |
| --- | --- | --- | --- | --- | --- | --- | --- | --- |
| Employee A (autism) |  |  |  |  |  |  |  |  |
| Employee B |  |  |  |  |  |  |  |  |
| Employee C |  |  |  |  |  |  |  |  |

3.8. What is the employee's approximate hourly rate of pay?

|  | $1-5 | $6-10 | $11-20 | $21-30 | $31-40 | $41-50 | $51-60 | $61+ | I do not know |
| --- | --- | --- | --- | --- | --- | --- | --- | --- | --- |
| Employee A (autism) |  |  |  |  |  |  |  |  |  |
| Employee B |  |  |  |  |  |  |  |  |  |
| Employee C |  |  |  |  |  |  |  |  |  |

3.9. What is the average overtime hours worked by employee per week (Overtime is work performed outside the ordinary hours listed in an award or agreement)?

|  | 0 | 1-5 | 6-10 | 11-15 | 16-20+ | I do not know |
| --- | --- | --- | --- | --- | --- | --- |
| Employee A (autism) |  |  |  |  |  |  |
| Employee B |  |  |  |  |  |  |
| Employee C |  |  |  |  |  |  |

3.10. What is the employee's approximate hourly rate of overtime pay?

|  | $10-30 | $31-50 | $51-70 | $71-90 | $91-100 | $110 | I do not know |
| --- | --- | --- | --- | --- | --- | --- | --- |
| Employee A (autism) |  |  |  |  |  |  |  |
| Employee B |  |  |  |  |  |  |  |
| Employee C |  |  |  |  |  |  |  |

3.11. Was there any additional training /staff development required for this worker once they

started work?

|  | Yes | No | I do not know |
| --- | --- | --- | --- |
| Employee A (autism) |  |  |  |
| Employee B |  |  |  |
| Employee C |  |  |  |

3.12. What type of additional training/staff development was required? (Multiple options allowed)

|  | Customer Service | Role specific skill re-inforcement/enhancement | Computer training | Conflict resolution | Behaviour management | Safety procedures | Sales strategies | Other |
| --- | --- | --- | --- | --- | --- | --- | --- | --- |
| Employee A  (autism) |  |  |  |  |  |  |  |  |
| Employee B |  |  |  |  |  |  |  |  |
| Employee C |  |  |  |  |  |  |  |  |

3.13. What type of "other" additional training/staff development was required?

3.14. Approximately how many hours of additional training/staff development has been required?

|  | 0 | 1-3 | 4-6 | 7-9 | 10-13 | 14-16 | 16+ |
| --- | --- | --- | --- | --- | --- | --- | --- |
| Employee A (autism) |  |  |  |  |  |  |  |
| Employee B |  |  |  |  |  |  |  |
| Employee C |  |  |  |  |  |  |  |

**Section 4: Employment Costs**

The purpose of this section is to collect information to use in estimating any additional employee costs to an organisation. These questions ask you to give answers on both an employee with autism in your organisation (employee A), as well as two matching employees without autism.

4.1. Is there any current or prior Workers’ Compensation claim for this employee?

|  | Yes | No | I do not know |
| --- | --- | --- | --- |
| Employee A (autism) |  |  |  |
| Employee B |  |  |  |
| Employee C |  |  |  |

4.2. Number of workers’ compensation claims because of injuries (yearly):

|  | 0 | 1-2 | 3-4 | 5-6 | 6+ | Not Applicable |
| --- | --- | --- | --- | --- | --- | --- |
| Employee A (autism) |  |  |  |  |  |  |
| Employee B |  |  |  |  |  |  |
| Employee C |  |  |  |  |  |  |

4.3. Have any changes to the workplace (eg. facilities) or job procedures been made for employees?

(Multiple options allowed)

|  | Ramps | Rails | Desks & Seating | Computer Adjustment | Lighting | Noise/  Sound  proofing | Barrier  isolation spaces | Equipment Adjustment | Matting Flooring | Other | None |
| --- | --- | --- | --- | --- | --- | --- | --- | --- | --- | --- | --- |
| Employee A (autism) |  |  |  |  |  |  |  |  |  |  |  |
| Employee B |  |  |  |  |  |  |  |  |  |  |  |
| Employee C |  |  |  |  |  |  |  |  |  |  |  |

4.4. Please provide details of what "other" changes to the workplace (eg. facilities) or job procedures was required for these employees:

4.5. Approximate total costs of the workplace changes:

|  | <$500 | $501-$1000 | $1001-$2000 | $2001-$5000 | $5001-  $10,000 | >  $10,000 | I do not know |
| --- | --- | --- | --- | --- | --- | --- | --- |
| Employee A (autism) |  |  |  |  |  |  |  |
| Employee B |  |  |  |  |  |  |  |
| Employee C |  |  |  |  |  |  |  |

4.6. Has this employee over the previous year had an unscheduled absence? *For example,* medical,

parental, bereavement, relocation, separation, or legal?

|  | Yes | No | I do not know |
| --- | --- | --- | --- |
| Employee A (autism) |  |  |  |
| Employee B |  |  |  |
| Employee C |  |  |  |

4.7. How many unscheduled absences occurred over the previous six months?

|  | 1-2 days | 3-4 days | 5-6 days | 6+ days | Not Applicable |
| --- | --- | --- | --- | --- | --- |
| Employee A (autism) |  |  |  |  |  |
| Employee B |  |  |  |  |  |
| Employee C |  |  |  |  |  |

4.8. Average amount of supervision required for each employee per week:

|  | 0 | 1-4 hours | 5-8 hours | 9-12 hours | 13-30 hours | 31-50 hours | 51+ hours | Not Applicable |
| --- | --- | --- | --- | --- | --- | --- | --- | --- |
| Employee A (autism) |  |  |  |  |  |  |  |  |
| Employee B |  |  |  |  |  |  |  |  |
| Employee C |  |  |  |  |  |  |  |  |

4.9. At your organisation approximately for how long has this employee with autism been

employed?

|  | > 36 months | 31-36 months | 25-30 months | 19-24 months | 13-18 months | 7-12 months | < 6 months |
| --- | --- | --- | --- | --- | --- | --- | --- |
| Employee A (autism) |  |  |  |  |  |  |  |
| Employee B |  |  |  |  |  |  |  |
| Employee C |  |  |  |  |  |  |  |

Thank you, we really appreciate your insight.

**Survey Feedback**

Were there any questions or topics in this survey missing that would have provided useful information?

Yes

No

If “yes”, what else would be good for us to know?

Additional feedback is gratefully received:

Would you be interested in participating in other studies?

Yes

No

If “yes”, please provide your contact information:

Name:

Email:

Mobile number:

Would you be interested in receiving a summary of the results of this study?

Yes

No

The results will be available in January 2016 and can be sent to you through email

Email:

***Thank you for taking the time to complete this survey. We value your insight and contribution to autism research.***
